# Postpartum continuous epidural analgesia on pain, sleep quality, and emotional state in childbearing women: a randomized controlled trial

**DOI:** 10.3389/fmed.2025.1582458

**Published:** 2025-09-17

**Authors:** Jialei Zhang, Jing Li, Xiaoling Zhang, Jie Wu

**Affiliations:** ^1^Department of Pain Treatment, Changzhi People’s Hospital Affiliated to Changzhi Medical College, Changzhi, China; ^2^Department of Basic Medical Sciences, Shanxi Medical University, Taiyuan, China; ^3^Department of Oncology, Changzhi People’s Hospital Affiliated to Changzhi Medical College, Changzhi, China

**Keywords:** pain, puerperium, epidural, sleep, depression

## Abstract

**Objective:**

This study aimed to explore the effects of continuous epidural analgesia on postpartum pain and sleep quality in postpartum women.

**Methods:**

First-time mothers who received labor analgesia in the obstetrics department of Changzhi People’s Hospital, affiliated to Changzhi Medical College, from November 2024 to December 2024, were selected and randomly divided into the control group (C group) and the experimental group (E group). The control group received epidural labor analgesia after entering the labor process, using 10 mL of 1% ropivacaine, 50 μg of sufentanil, and 90 mL of 0.9% sodium chloride injection. The initial bolus dose was 6 mL, the injection rate was 6 mL/h, with additional doses of 4 mL each time, a lockout time of 15 min, and continuous infusion until the fetus was delivered and then stopped. The experimental group received epidural labor analgesia after entering the labor process and continued the infusion for 2 days after the fetus was delivered, with the same medication concentration and infusion rate as the control group. All postpartum mothers had blood samples taken before sleep on the 1st and 3rd days after delivery to measure serum melatonin levels. The visual analog scale (VAS) was used to compare pain scores (including sitting pain, sleep pain, and urination pain) on the 1st and 3rd days after the fetus was delivered in both groups. The modified Bromage score (MBS) was used to evaluate the mobility of the lower limbs. On the 3rd postoperative day, the Edinburgh Postnatal Depression Scale (EPDS) and the Pittsburgh Sleep Quality Index (PSQI) were used to assess sleep quality and emotional state, and the occurrence of depression in both groups.

**Results:**

The VAS scores for sitting pain, sleep pain, and urination pain on the 1st and 3rd days after the fetus was delivered were all lower in the experimental group compared to the control group (*p* < 0.05). There was no significant difference in MBS between the two groups on the first day after delivery (*p* > 0.05), and the experimental group had higher scores than the control group on the 3rd day (*p* < 0.05), but this did not significantly affect the patients’ daily lives in the experimental group. In addition, the EPDS and PSQI scores were both higher in the experimental group than in the control group (*p* < 0.05). The levels of melatonin in the body were also significantly higher in the experimental group than the control group (*p* < 0.05).

**Conclusion:**

The implementation of continuous epidural analgesia after childbirth can significantly alleviate postpartum pain, improve sleep quality, and reduce the occurrence of postpartum depression without affecting the mother’s mobility.

## Introduction

Labor pain is the intense sensation of pain caused by the strong contractions of the uterus during the first stage of labor and the compression and expansion of the vagina and perineum by the fetus during the second stage (1). In addition, soft tissue damage and the occurrence of local inflammatory responses during childbirth can also lead to the development of chronic postpartum pain (2). Perinatal pain may, on the one hand, cause significant changes in the hemodynamics of the parturient, leading to complications such as eclampsia and convulsions (3), and, on the other hand, may trigger negative emotions such as anxiety and depression in the mother (4), leading to sleep disturbances, affecting the recovery prognosis and the quality of life postpartum.

The currently accepted method of labor analgesia with the least impact on both the mother and the fetus is neuraxial anesthesia, including continuous epidural blockade and combined spinal-epidural anesthesia. However, medication is often discontinued after the fetus is delivered, thereby neglecting the management of postpartum pain. Studies show that acute perineal pain after vaginal delivery is common, with a significantly higher incidence in primiparous women compared to multiparous women. In addition, breastfeeding may lead to increased secretion of oxytocin, causing uterine contractions and resulting in postpartum spasmodic pain (5). Extending epidural analgesia beyond delivery may provide segmental, opioid-sparing analgesia and—by reducing pain—secondarily improve sleep and emotional state. The severity of acute postpartum pain predicts persistent pain and postpartum depression, and effective labor analgesia has been associated with lower depressive symptom scores. However, randomized evidence on continuing epidural infusion into the postpartum period—and its effects on sleep, mood, and melatonin—remains limited.

Accordingly, we conducted a randomized controlled trial in nulliparous women to compare continuous epidural analgesia maintained for 48 h after delivery with discontinuation at delivery. Outcomes included visual analog scale (VAS) pain during common activities (sitting, sleeping, urination) on postpartum days 1 and 3; sleep quality on day 3 using the Pittsburgh Sleep Quality Index (PSQI); depressive symptoms on day 3 using the Edinburgh Postnatal Depression Scale (EPDS); and nocturnal serum melatonin measured before sleep (22:00–23:00) on postpartum days 1 and 3. Safety was assessed by the modified Bromage score (MBS) for lower limb motor block and by adverse events. We hypothesized that postpartum continuous epidural analgesia would reduce pain, improve sleep quality, and lower depressive symptoms, be associated with higher nocturnal melatonin, and maintain acceptable maternal mobility and safety.

## Methods

### Study design

This study is a randomized controlled trial. By comparing the pain scores, sleep quality, emotional state, and serum melatonin levels of postpartum women who received continuous epidural analgesia with those who did not, we aim to investigate the impact of continuous epidural analgesia after childbirth on postpartum women. The experiment has been approved by the Medical Ethics Committee of Changzhi City People’s Hospital, all experiments were conducted in accordance with relevant guidelines and regulations, and it is registered with the Chinese Clinical Trial Registration Center (Clinical Trial Number: ChiCTR2400091663, 31 October 2024). All participating patients have signed informed consent forms.

### Patients

Participants were selected from patients aged 25 to 35, with singleton pregnancies, cephalic presentation, and full-term nulliparous women who were scheduled for labor analgesia at the Obstetrics Department of Changzhi City People’s Hospital from November 2024 to December 2024. Exclusion criteria included history of mental or psychological disorders; preeclampsia; organic diseases or functional disorders of important organs such as the heart or brain; fetal malformations; and spinal deformities or infections at the puncture site.

### Randomization and allocation concealment

We used simple randomization with a 1:1 allocation ratio implemented via sequentially numbered, opaque, sealed envelopes (SNOSE). An independent study coordinator (not involved in participant recruitment, intervention delivery, or outcome assessment) prepared an equal number of allocation cards labeled “Experimental (E group)” and “Control (C group),” thoroughly shuffled them, and sealed them into tamper-evident opaque envelopes pre-numbered in sequence. After informed consent and eligibility confirmation, the enrolling clinician opened the next envelope in sequence. Allocation was revealed prior to epidural catheter management to ensure protocol adherence. Enrolling investigators could not access or predict allocations before opening the envelope.

### Sample size and power

We calculated the sample size for a randomized controlled design with a binary endpoint (occurrence of a pain event). Assuming a two-sided *α* = 0.05 and power 1 − *β* = 0.90, a pilot estimate suggested a control-group event rate *P*2 = 0.80. To achieve a clinically important effect, we targeted a 50% relative risk reduction with the new therapy, i.e., risk ratio R = *P*1/*P*2 = 0.50 and experimental group event rate p_1_ = 0.40. Using the two-proportion formula,


$$n=\frac{10.51[(R+1)-P2(R^{2}+1)]\;}{P2(1-R)}$$

the required per-group sample size is *n* ≈ 26.3; thus, we planned for at least *n* ≥ 27 participants per group (total ≥ 54). If accounting for approximately 10–15% attrition, the target would be 30–32 per group.

### Pain management plan

All parturient women were connected to cardiac monitoring upon entering the delivery room, and fetal heart rate was continuously monitored to determine whether to terminate spontaneous labor. The control group underwent routine continuous epidural analgesia for painless childbirth. After the women entered the labor process, epidural analgesia was performed with a puncture at the L2-3 interspace, and a catheter was placed. A test dose of 3 mL of 2% lidocaine was injected into the epidural space, and after observing for 5 min without any adverse reactions in the parturient, the epidural analgesia pump was connected. The analgesia pump was prepared with 10 mL of 1% ropivacaine, 50 μg of sufentanil, and 90 mL of 0.9% sodium chloride injection. The initial bolus dose was 6 mL, and the injection rate was 6 mL/h, with additional doses of 4 mL each time, a lockout time of 15 min, and continuous infusion until the fetus was delivered and then stopped. The experimental group underwent epidural analgesia at the L2-3 interspace after entering the labor process, with continuous infusion until 2 days after the fetus was delivered, with the same medication concentration and infusion rate as the control group.

### Observational indicators

The basic information of the patients was recorded, including age, body mass index, history of hypertension, history of diabetes, total duration of labor, and postpartum blood loss. Blood samples were taken from all parturient women before sleep on the 1st and 3rd days after delivery to measure serum melatonin levels. The visual analog scale was used to compare pain scores (including sitting pain, sleep pain, and urination pain) on the 1st and 3rd days after the fetus was delivered in both groups. The modified Bromage score was used to evaluate the degree of motor block of the lower limbs in patients. On the 3rd postoperative day, the Pittsburgh Sleep Quality Index (PSQI) and the Edinburgh Postnatal Depression Scale (EPDS) were used to assess sleep quality and emotional state in both groups and to determine whether depression occurred.

- Primary outcomes (pre-specified)

- Postpartum pain intensity on day 3: VAS (0–10) for sitting pain, sleep pain, and urination pain.
- Sleep quality on day 3: Pittsburgh Sleep Quality Index (PSQI; total score 0–21; lower scores indicate better sleep), also summarized by standard PSQI categories.

- Secondary outcomes
- VAS pain on day 1 (sitting pain, sleep pain, urination pain).
- Depressive symptoms on day 3: Edinburgh Postnatal Depression Scale (EPDS; total 0–30; lower scores indicate fewer depressive symptoms), summarized by EPDS categories.
- Serum melatonin levels on day 1 and day 3 (blood drawn before sleep, 22:00–23:00).
- Lower-limb motor block on day 1 and day 3: modified Bromage score (0: none; 1: partial; 2: almost complete; 3: complete; lower is better).
- Safety: postpartum nausea, vomiting, dizziness, hypotension, constipation, and urinary tract infection.

### Psychometric properties of outcome measures

The VAS is a unidimensional measure of acute pain intensity that shows strong test–retest reliability, good convergent validity with numeric/verbal rating scales, and high responsiveness to perioperative pain change; contemporary reviews also support interpreting between-group differences in relation to minimal clinically important differences (MCID), commonly approximating 1–2 points for acute pain contexts, which we prespecified to aid clinical interpretability of our findings (6). In this trial, assessors were trained with standardized instructions and identical timing across postpartum days 1 and 3 to minimize rater and context variability. The 10-cm Visual Analog Scale with Chinese anchor terms (“no pain” to “worst pain”) demonstrates good reliability and convergent validity in clinical contexts, supporting its use for pain intensity assessment in Chinese populations (7).

The modified Bromage score (MBS) is an observer-rated ordinal scale widely used to quantify lower-limb motor block in neuraxial anesthesia and labor analgesia. It offers high feasibility at the bedside, clear gradations with face validity for functional mobility, and supports standardized safety monitoring in obstetric anesthesia trials (8). In this study, raters followed a written protocol and assessed patients in a consistent position and time window to improve reproducibility across postpartum days 1 and 3 (0 points, no motor blockade; 1 point, partial blockade, unable to raise the straight leg, but the knee can be bent; 2 points, almost complete blockade, the knee cannot be bent, but the ankle joint can move; 3 points, complete blockade, no movement). Recent studies demonstrate good operability and reproducibility, supporting its use for standardized motor-block assessment in Chinese parturients receiving neuraxial anesthesia (9).

The Pittsburgh Sleep Quality Index (PSQI) questionnaire assesses sleep quality based on sleep quality, sleep duration, sleep latency, sleep efficiency, number of nighttime awakenings, and daytime dysfunction, with a score ranging from 0 to 21 points. A score of 0 to 5 indicates very good sleep quality, 6 to 10 indicates good sleep quality, 11 to 15 indicates fair sleep quality, and 16 to 21 indicates poor sleep quality (10). The Chinese version of the PSQI demonstrates acceptable-to-good internal consistency and a stable factor structure in Chinese samples, supporting its use for screening and research; its multidimensional design provides sensitivity to clinically relevant changes in sleep quality, which underpins our use of the total score and standard categorical bands to summarize outcomes on postpartum day 3. We used the validated Chinese PSQI, administered within a fixed nocturnal window to control for circadian and environmental influences (11).

The Edinburgh Postnatal Depression Scale (EPDS) is used for the assessment of depression, evaluating three aspects: emotional loss, anxiety, and depression. The total score ranges from 0 to 30 points, with 0 to 8 points indicating normal levels, 9 to 13 points indicating a high-risk group, and scores above 13 suggesting postpartum depression (12). Recent large-scale studies in Chinese perinatal populations confirm a robust three-factor structure (anhedonia, anxiety, depression), good internal consistency, and longitudinal measurement invariance across pregnancy and postpartum, supporting both cross-sectional comparisons and temporal stability of the construct measured by the Chinese EPDS (13). These findings justify our use of the EPDS total score, alongside standard risk strata, to quantify depressive symptoms at postpartum day 3 and to compare groups without bias from factor instability or differential item functioning across time.

### Statistical analysis

Data analysis was performed using the SPSS Statistics 23.0 software. The Shapiro–Wilk test was used to determine whether each set of data was normally distributed. Normally distributed quantitative data were expressed as the mean ± standard deviation ($\bar{x\;}\;$ ± s), and inter-group comparisons were analyzed using the independent samples *t*-test. Intra-group comparisons were performed using repeated measures analysis of variance. Categorical data were expressed as medians and interquartile ranges, and numerical data were presented as counts and percentages. Categorical variables were analyzed using chi-square tests, chi-square tests with continuity correction, or Fisher’s exact tests. Non-normally distributed quantitative data were expressed as medians (M) and interquartile ranges (IQR), and inter-group comparisons were made using the Mann–Whitney U test. A *p*-value of less than 0.05 was considered statistically significant.

## Results

### Patient recruitment

**Figure 1:**
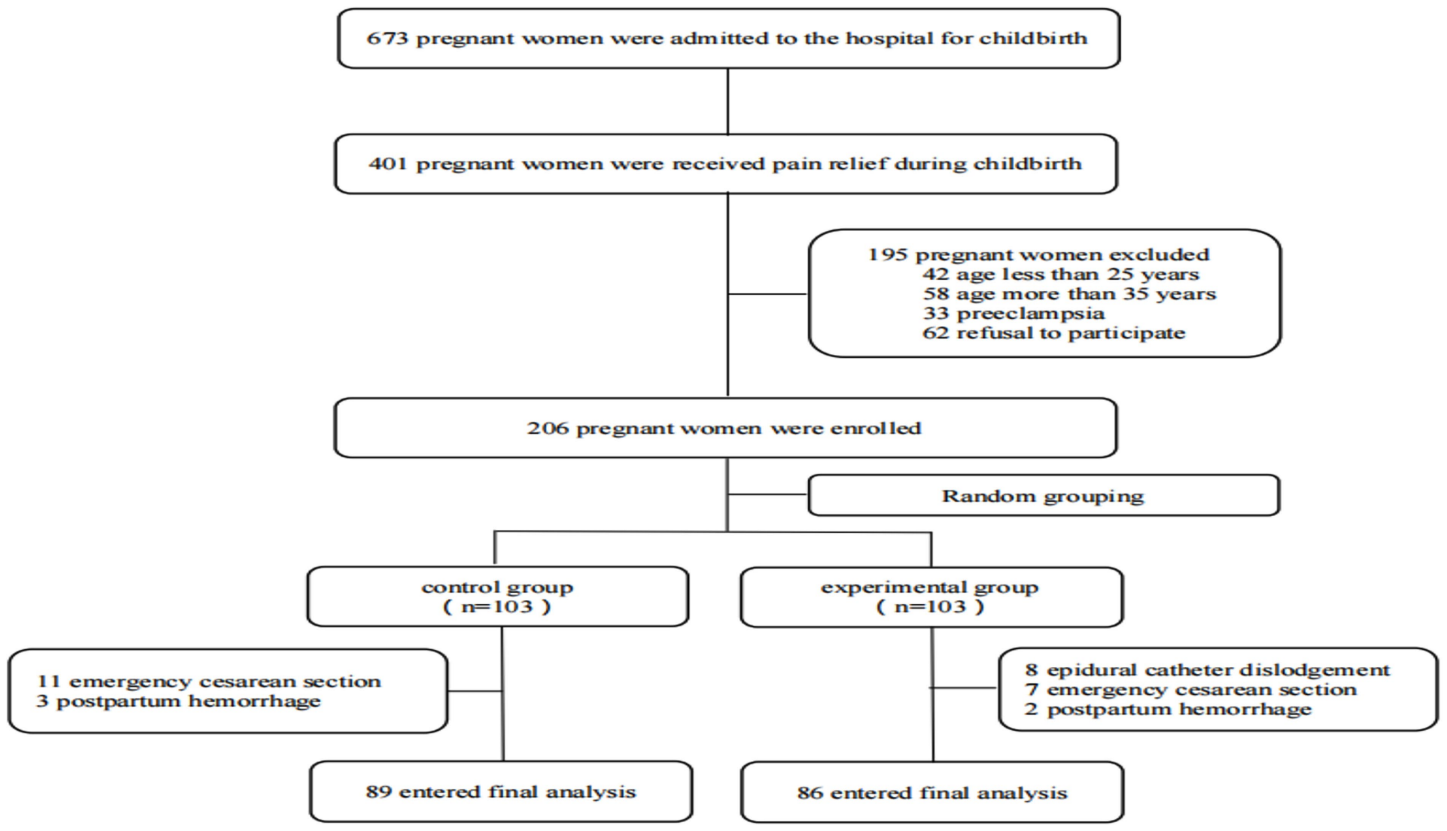
Flow chart of the study.

From November 2024 to December 2024, a total of 673 parturients were admitted to our hospital’s obstetrics department, among whom 401 received labor analgesia. Out of these, 206 met the inclusion criteria, and ultimately, 175 were enrolled and completed the entire trial. Randomization was performed using the SPSS software and a random number table, dividing the participants into a control group (C group) and an experimental group (E group). There were no significant differences in age, body mass index, history of hypertension, history of diabetes, total duration of labor, and postpartum blood loss between the two groups (*p* > 0.05) (Figure 1; Table 1).

**Table 1:** Demographic and baseline characteristics.

Group	Age (years)	BMI (kg/m^2^)	Hypertension (*n*, %)	Diabetes (*n*, %)	Stage of labor (min)	Amount of bleeding (mL)
C group (*n* = 89)	27.68 ± 2.73	25.70 ± 1.37	17	27	393.28 ± 58.28	183.46 ± 24.27
E group (*n* = 86)	26.98 ± 2.66	25.17 ± 1.30	21	22	379.22 ± 56.48	196.54 ± 19.02
*t*/χ^2^	1.299	0.368	0.728	0.491	0.876	0.826
*P*	0.197	0.713	0.394	0.484	0.383	0.411

### Pain score comparison

**Figure 2:**
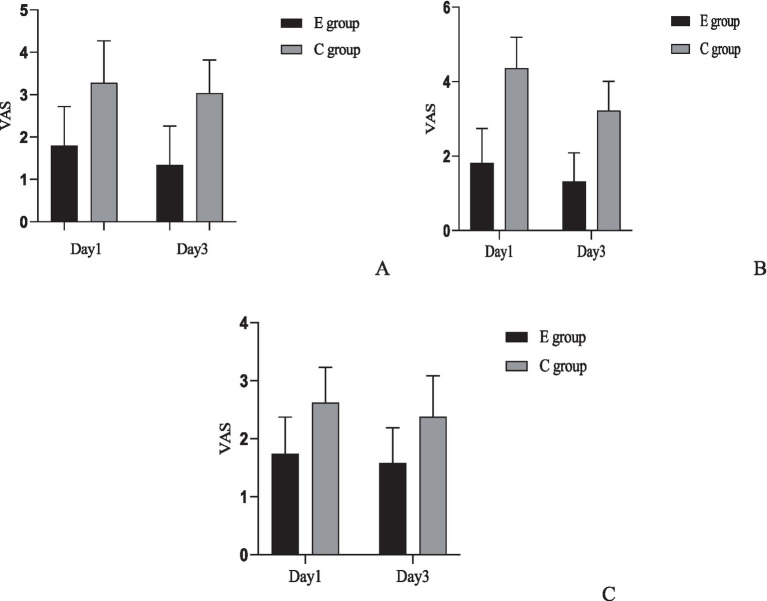
Comparison of pain scores between the two groups. **(A)** Comparison of sitting pain VAS scores between the two groups: Inter-group comparison: Day 1: *t* = 7.718, *p* < 0.001; Day 3: *t* = 9.976, *p* < 0.001. Intra-group comparison: C group: *t* = 1.345, *p* = 0.182; E group: *t* = 2.496, *p* = 0.014. **(B)** Comparison of urination pain VAS scores between the two groups: Inter-group comparison: Day 1: *t* = 14.527, *p* < 0.001; Day 3: *t* = 12.196, *p* < 0.001. Intra-group comparison: C group: *t* = 7.048, *p* < 0.001; E group: *t* = 2.953, *p* = 0.004. **(C)** Comparison of sleep pain VAS scores between the two groups. Inter-group comparison: Day 1: *t* = 7.122, *p* < 0.001; Day 3: *t* = 6.113, *p* < 0.001. Intra-group comparison: C group: *t* = 1.843, *p* = 0.068; E group: *t* = 1.288, *p* = 0.201.

A comparison of the VAS scores for sitting pain, sleep pain, and urination pain was conducted between the two groups. Inter-group comparison results indicated that the experimental group had lower pain scores for all categories on the 1st and 3rd postpartum days compared to the control group, with statistically significant differences (*p* < 0.05). For intra-group comparisons, the control group showed improvement in urination pain on the 3rd day (*p* < 0.05), but no significant differences were observed in sitting pain and sleep pain compared to the 1st day (*p* > 0.05). In the experimental group, improvements were noted in sitting pain and urination pain (*p* < 0.05), while sleep pain showed no significant difference compared to the 1st day (*p* > 0.05) (Figure 2).

We added effect size and confidence interval reporting for all primary comparisons regarding VAS pain scores. For each time point and pain type, between-group mean differences were calculated along with 95% confidence intervals (CIs), and Cohen’s d effect size with its 95% CI was computed. On postpartum day 1, the mean difference in sitting pain VAS (E group minus C group) was −1.48 (95% CI: −1.77 to −1.19, Cohen’s d = −1.55, 95% CI: −1.89 to −1.21); for urination pain −2.54 (95% CI: −2.80 to −2.28, d = −2.90, 95% CI: −3.33 to −2.48); and for sleep pain −0.88 (95% CI: −1.06 to −0.70, d = −1.43, 95% CI: −1.76 to −1.10). On postpartum day 3, differences remained significant: sitting pain −1.70 (95% CI: −1.95 to −1.45, d = −2.00, 95% CI: −2.36 to −1.63); urination pain −1.90 (95% CI: −2.13 to −1.67, d = −2.44, 95% CI: −2.83 to −2.04); and sleep pain −0.80 (95% CI: −1.00 to −0.60, d = −1.22, 95% CI: −1.54 to −0.89). All effect sizes indicate large between-group effects. *p*-values presented were not adjusted for multiple comparisons.

We additionally assessed within-group changes in VAS pain scores between postpartum day 1 and day 3 for each group. In the E group, sitting pain VAS decreased by a mean of 0.46 points (95% CI: 0.18 to 0.74, Cohen’s d = 0.50, 95% CI: 0.28 to 0.72), urination pain by 0.50 points (95% CI: 0.24 to 0.76, d = 0.59, 95% CI: 0.36 to 0.82), and sleep pain by 0.16 points (95% CI: −0.03 to 0.35, d = 0.26, 95% CI: 0.04 to 0.47). In the C group, sitting pain decreased by 0.24 (95% CI: −0.03 to 0.51, d = 0.27, 95% CI: 0.06 to 0.48), urination pain by 1.14 (95% CI: 0.90 to 1.38, d = 1.41, 95% CI: 1.11 to 1.70), and sleep pain by 0.24 (95% CI: 0.05 to 0.43, d = 0.37, 95% CI: 0.15 to 0.58). All *p*-values were not adjusted for multiple comparisons.

### Comparison of lower extremity motor blockade severity

The results showed that there was no significant difference in MBS scores between the two groups on the first day (*p* > 0.05), but on the third day, the lower extremity blockade score of patients in the E group was significantly lower than that in the C group (*p* < 0.05), which did not affect the patients’ daily life. At the same time, when comparing within the groups, there was no significant difference in the E group (*p* > 0.05), while the score of the C group on the third day was significantly lower than on the first day (*p* < 0.05) (Table 2).

**Table 2:** Comparison of lower extremity mobility between the two groups.

Group	Day 1	Day 3	*z*	*p*
C group	1.0 (0.0–1.0)	0.0 (0.0–0.0)	2.687	0.007
E group	1.0 (0.0–1.0)	1.0 (0.0–1.0)	1.855	0.064
*z*	1.428	2.283		
*p*	0.153	0.022		

### Comparison of melatonin levels

**Figure 3:**
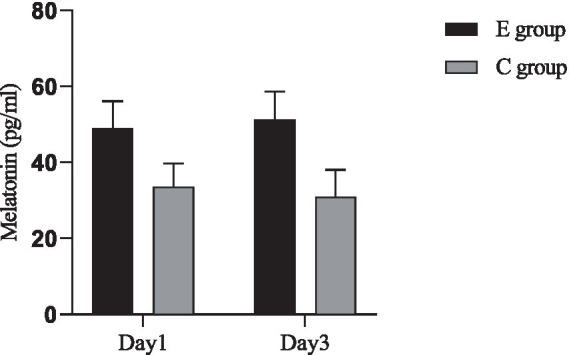
Comparison of melatonin levels between the two groups. Inter-group comparison: Day 1: *t* = 11.616, *p* < 0.001; Day 3: *t* = 13.973, *p* < 0.001. Intra-group comparison: C group: *t* = 2.032, *p* = 0.045; E group: *t* = 1.524, *p* = 0.131.

A comparison of serum melatonin levels between the two groups was conducted. The inter-group comparison showed that the melatonin levels in group E were significantly higher than those in group C, with a statistically significant difference (*p* < 0.05). For intra-group comparisons, no significant difference was observed in group E (*p* > 0.05), while in group C, the levels on the 3rd day were significantly lower than on the 1st day (*p* < 0.05) (Figure 3).

### Comparison of PSQI and EPDS scores

The PSQI and EPDS scores of two groups of patients were compared. The results showed that there were significantly more patients with low PSQI and EPDS scores in Group E than in Group C, with statistically significant differences (*p* < 0.05). This indicates that the sleep quality and emotional state of parturients in Group E were significantly better than those in Group C (Table 3).

**Table 3:** Comparison of postpartum PSQI and EPDS scores between the two groups of parturients.

Group	PSQI (*n*)				EPDS (*n*)		
	0~5	6~10	11~15	16~21	0~8	9~13	>13
C group (*n* = 89)	8	20	34	27	27	51	11
E group (*n* = 86)	26	39	16	5	52	31	3
χ^2^	37.213				17.315		
*P*	<0.001				<0.001		

### Comparison of adverse reactions

There was no significant difference in the incidence of postpartum nausea, vomiting, dizziness, hypotension, and urinary tract infection between the two groups of parturients (*p* > 0.05), while the number of patients with constipation in the C group was significantly higher than that in the E group (*p* < 0.05) (Table 4).

**Table 4:** Statistical comparison of adverse reactions in two groups of parturients.

Complication	C group	E group	*p*
Nausea (*n*, %)	7	6	0.823
Vomit (*n*, %)	3	3	0.966
Dizzy (*n*, %)	5	3	0.720
Hypotension (*n*, %)	4	6	0.531
Constipation (*n*, %)	9	1	0.018
Urinary tract infection (*n*, %)	2	0	0.497

## Discussion

Postpartum period pain is one of the most common complaints among women after childbirth, with over 90% of women who deliver vaginally experiencing varying degrees of perineal pain during the puerperal period (14) and 47% of women enduring intense uterine contraction pain within 48 h postpartum (15). The causes of postpartum pain are often considered to be soft tissue damage, nerve damage, and local inflammatory responses that lead to peripheral and central nerve sensitization (16). Poorly controlled pain can have adverse physiological and psychological effects on patients, not only prolonging hospital stays and recovery times but also increasing the incidence of acute pain transitioning into chronic pain. Postpartum pain not only severely affects the physical and mental health of women who have just given birth but may also lead to negative emotions such as tension, anxiety, and irritability. Moreover, it can excessively activate the sympathetic nervous system, promoting the secretion of adrenal cortical hormones and causing drastic changes in hemodynamics and blood glucose levels (17). Therefore, there is still a significant demand for treatment and management of postpartum pain to mitigate its impact on women who have recently given birth.

Many studies have shown that multimodal analgesia helps to reduce postpartum pain. Due to its minimal transfer into breast milk and excellent safety profile, acetaminophen is generally the first-choice analgesic for postpartum pain (18). A clinical randomized controlled study comparing the different effects of two multimodal analgesic regimens on acute pain after cesarean section found that oral acetaminophen can reduce the risk of acute postoperative pain, and acetaminophen has become the top choice for many obstetricians in treating postpartum pain (19). However, more and more experimental and epidemiological studies have shown that prenatal exposure to acetaminophen may affect fetal development, potentially increasing the risk of fetal nervous system development and urogenital system abnormalities, and there is no evidence that this risk will not affect fetal development through breast milk, which greatly limits the clinical application of acetaminophen (20, 21). Considering the above factors, combined with the observation that the incidence of complications in the observation group of this trial was not significantly different from the control group, postpartum self-controlled epidural analgesia can be seen as effective and satisfactory pain relief for postpartum women, just like labor analgesia. The results of this study show that the VAS scores for sitting pain, sleep pain, and urination pain on the 1st and 3rd postpartum days in the experimental group were significantly lower than those in the control group, showing a clear analgesic effect. Moreover, a small number of patients in the control group had significant sitting pain and urination pain, which led to resistance to urination and defecation, resulting in constipation and urinary tract infection. Considering all factors, it can be concluded that continuous epidural analgesia can be an effective pain relief method for postpartum women, reducing their pain, improving their quality of life, and enhancing treatment satisfaction.

Pain and poor sleep quality often coexist (22), with insufficient sleep leading to increased pain sensitivity and pain altering sleep architecture. Melatonin is a hormone produced by the pineal gland and is believed to be related to the sleep–wake cycle. It can improve postoperative sleep quality through antioxidant and immune-modulating effects, and its level changes are considered one of the markers of sleep disorders (23). Animal studies have shown that sleep disorders can upregulate the activation of pro-inflammatory factors in the hippocampus of mice, suppressing melatonin secretion (24), while exogenous melatonin can improve sleep disorders and enhance sleep quality by affecting the circadian rhythm (25). The latest research shows (26) that, in addition to improving sleep disorders, melatonin can also reduce the use of analgesics in patients with knee/hip joint inflammation and alleviate pain behaviors in rats by increasing serum glycine levels. Since the synthesis and release of melatonin by the pineal gland are related to the circadian rhythm, this study chose to collect blood from the parturients before sleep (between 22:00 and 23:00 at night) to detect the levels of melatonin in the body. The results of this trial show that the levels of melatonin and sleep quality in the experimental group were significantly higher than those in the control group, similar to previous results that painless childbirth technology can significantly improve the sleep quality of parturients (27), indicating that continuous epidural analgesia technology can also significantly improve the sleep quality of parturients during the puerperal period. This may be due to the painless technology reducing the pain index of parturients while alleviating their emotional stress, making it easier for them to relax and fall asleep. The results of this study also found a new issue: Although the pain level of the control group on the third day after delivery was somewhat relieved compared to the first day, the level of melatonin did not increase similarly to the experimental group but instead showed a more significant decrease. It is considered that the impact of pain on the pineal gland may be more prolonged and did not ease with the reduction of pain stimulation. The specific mechanism awaits further research.

In non-obstetric populations, the comorbidity between pain and depression has been confirmed. Epidemiological studies have shown that the incidence of neuropathic pain in patients with depression is six times that of the general population, while the incidence of depression in patients with pain is 3 to 5 times that of pain-free individuals, and this number continues to increase with the severity of pain (28). In recent years, the severity of pain has also been proven to be closely related to the occurrence of postpartum depression in women. Studies have shown that acute pain after childbirth can serve as an important risk predictor for persistent, chronic pain and postpartum depression, with no significant difference related to the mode of delivery (29). Despite substantial evidence confirming that effective analgesia during childbirth can reduce the risk of postpartum depression, few studies have addressed the relationship between postpartum pain management and postpartum depression. Studies have indicated that postpartum pain is an important factor in the Edinburgh Postnatal Depression Scale (EPDS) scores, and improvement in pain severity can reduce EPDS scores. However, the majority of mothers restrict the use of analgesic drugs due to concerns about the potential impact on infant development through breast milk (30). Therefore, the advantages of epidural analgesia become apparent. The results of this trial show that the EPDS depression scores in the observation group were significantly better than those in the control group. Combining the pain scores of both groups, it is believed that pain relief not only allows the physical relaxation of the parturient but also alleviates their psychological stress, preventing negative emotions such as anxiety and irritability caused by pain. At the same time, we also found that even with adequate analgesia, there were still three suspected cases of depression in the experimental group, suggesting that pain may be a high-risk factor for postpartum depression but not the only one. Recent studies show that, in addition to pain factors, factors such as age, educational status, employment status, history of depression, and the condition of the newborn are all correlated with postpartum depression (31).

This study did not find any complications related to spinal puncture, but the possibility of complications occurring should still be vigilantly monitored. Studies have shown that spinal anesthesia is an independent risk factor for postpartum low back pain, which may be related to the stimulation and injury of the ligaments, blood vessels, and nerves of the parturient during lumbar puncture, as well as postoperative pelvic infections and other reasons (32). Therefore, while providing effective analgesia during the puerperal period, it is also necessary to continuously pay attention to the occurrence of related complications and to handle them promptly. Meanwhile, this randomized trial was conducted at a single tertiary center with locally standardized obstetric and analgesia pathways, which may limit generalizability to other settings and populations. Follow-up was restricted to the early puerperium (through postpartum day 3), precluding assessment of durability and later outcomes (e.g., persistent pain, functional recovery, breastfeeding, and mood at 6 weeks).

### Limitations

Although this study concluded that continuous epidural analgesia can improve postpartum pain, sleep quality, and emotional state, the following limitations remain: This trial did not collect antenatal (late-pregnancy or pre-delivery) baseline measures of sleep quality (PSQI), depressive symptoms (EPDS), or nocturnal serum melatonin. Consequently, we cannot fully exclude the possibility that unmeasured antenatal differences in sleep, mood, or circadian biology contributed to the observed postpartum between-group differences. Although randomization, identical intrapartum analgesic regimens across groups, balanced clinical characteristics at baseline (Table 1), and standardized postpartum assessment time points mitigate confounding to some extent, residual confounding remains possible. Because all participants had been discharged and the approved consent did not include post-discharge back-collection of baseline questionnaires or biomarkers, these data cannot be supplemented retrospectively. Future studies will obtain antenatal (or admission) PSQI, EPDS, and nocturnal (22:00–23:00) serum melatonin as baseline covariates in a preregistered analysis plan, extend follow-up to 6–12 weeks postpartum, and consider objective sleep metrics (e.g., actigraphy) to further test the robustness of our findings.

## Conclusion

In conclusion, continuous epidural analgesia after childbirth has a positive significance in managing postpartum pain. When used without significant complications, it can help alleviate negative emotions in new mothers, enhance sleep quality, and facilitate postoperative recovery. This approach offers a new option for reducing the pain experienced by women during the puerperal period.

## Data Availability

The raw data supporting the conclusions of this article will be made available by the authors, without undue reservation.
